# Demographic Profile and Etiology of Epistaxis

**DOI:** 10.1007/s12070-024-04638-3

**Published:** 2024-04-06

**Authors:** Lavi Ukawat, Ankur Gupta, Konika Jain

**Affiliations:** 1 ENT Department, GMC, Haldwani, Uttarakhand India; 2 ENT Department, SRMS IMS, Bareilly, UP India; 3 Community medicine department, Gov. Medical College, Ratlam, MP India

**Keywords:** Epistaxis, ENT emergency, Etiology

## Abstract

Epistaxis is common worldwide otorhinolaryngology emergency presenting as a life-threatening condition especially in resource-constrained hospitals with limited health-care facilities for acceptable management. The aim of this study is to find out the common causes of epistaxis. It was a cross sectional study. It was carried out on 304 patients who presented with epistaxis at tertiary care hospital of Central India (Peoples College of Medical Science & Research Centre, Bhopal). It was found that among 304 participants, maximum number of patients with epistaxis were of age group 21–30 years i.e. 66 (21.71%) with 210 (69.08%) were male and 94 (30.92%) were female. It was found that maximum patients were of nose picking i.e. 113 (37.17%) followed by trauma via accident, assault and fall i.e. 77 (25.33%), followed by hypertension i.e. 49. Epistaxis is a common emergency condition in Otorhinolaryngology. People of all ages can be affected. Hypertension and trauma were the most common etiological/risk factors among the patients in whom etiology was found although in most of the patients etiology could not be found.

## Introduction

Epistaxis is defined as the bleeding from inside the nose or nasal cavity. It is one of the most common emergencies in Otorhinolaryngology worldwide which often requires admission to the hospital [1]. Its incidence is difficult to assess but it is expected that approximately 60% of the population will be affected by epistaxis at some point in their lifetime, with 6% requiring medical attention [2]. It is common worldwide otorhinolaryngological emergency presenting as a life-threatening condition especially in resource-constrained hospitals with limited health-care facilities for acceptable management [3]. Both genders and all the age groups commonly affected but young males usually are more affected as a result of a larger experience to trauma [4]. The etiological factors in epistaxis are diverse and are classified as either local, i.e., resulting from damage to the nasal epithelial mucosal lining or systemic with some studies reporting another group referred to as idiopathic in which a cause cannot be ascribed [5]. Nasal packing still represents the first-line approach to epistaxis in most emergency room, the gelatin-thrombin matrix as the better material and high cost-effectiveness of the management of posterior epistaxis than ribbon gauze or the Rapid Rhino [6]. The aim of this study is to find out the common causes of epistaxis.

## Methodology

It was a cross sectional study. The study has been carried out on patients who presented with epistaxis at tertiary care hospital of Central India (Peoples College of Medical Science & Research Centre, Bhopal). We took all patients as per case selection criteria who presented with epistaxis during the study period. Patients of both sexes and all age groups who presented with epistaxis were included in this study. Patients who presented with epistaxis resulting from recent septal or paranasal sinus surgery (within 3 weeks) and those not giving the consent for the study were excluded from this study. A total of 304 study participants were included.

## Data Collection Method

Each respondents were explained in brief about the need and purpose of the study; and ascent & consent for study was taken from patients and guardian of the pediatric patients. All the patients who presented with epistaxis at People’s Medical Hospital were evaluated completely, after taking detailed history followed by complete General, Local and ENT examination, diagnostic and treatment evaluation. Information was collected through using pre designed and pre tested proforma. Data was entered in Microsoft excel 2007 and analyzed using Epi info 7. Numerical variables was described in mean and standard deviation or median and inter-quartile range. Categorical variables was described in count and proportion at 95% confidence interval. All variable were analyzed using Chi square test of significance; *P* < 0.05 was taken as statically significant.

## Result

### Age Distribution

Among 304 participants, maximum number of patients with epistaxis were of age group 21–30 years i.e. 66 (21.71%) and least number of patients were of age group 71–80 years and 81–90 years i.e. 1 (0.33%). In this study, patients within the age group of 0–30 years consists of 62.5% of patients of epistaxis.

### Sex Distribution

Total study patients taken were 304, out of which 210 (69.08%) were male and 94 (30.92%) were female. The male to female ratio was found to be 2.23.

The maximum patients were male in age group 11–20 years i.e. 50, followed by 41 which were of age group 1–10 years and in female, maximum were of age group 21–30 years i.e. 28, followed by 21 of age group 1–10 years as seen in Table 1.

**Table 1:** Age and gender wise distribution of study participants

Age Group(yrs)	Gender		Total
	Male	Female	
< 10	41	21	62(20.3%)
11–20	50	12	62(20.3%)
21–30	38	28	66(21.7%)
31–40	29	13	42(13.8%)
41–50	28	11	39(12.8%)
51–60	17	6	23(7.5%)
61–70	6	2	8(2.6%)
71–80	0	1	1(0.3%)
81–90	1	0	1(0.3%)
Total	210	94	304(100.0%)

### Seasonal Distribution

It was found that maximum patients of epistaxis in summer season i.e. 143 (47.04%) followed by winter i.e. 104 (34.21%) and least in monsoon i.e.20 (6.58%).

### Duration and Episode of Bleeding

The maximum patients came with duration of nasal bleed of between 2 and 15 days that is 137 patients (45.07%) followed by less than one day duration in 135 patients (44.41%), and minimum that is 11 (3.62%) patients came with duration of nasal bleed more than 180 days. Out of total 304 patients of epistaxis, 206 (67.7%) patients had only single episode of epistaxis while 98 (32.24%) patients had recurrent episodes of epistaxis.

### Side, Type and Amount of Bleed

Among total 304 patients, 135 (44.41%) patients had bleed from right nostril and 28 (9.21%) had bleed from left nostril and 141 from bilateral which was highest i.e.46.38%. Maximum patients according to involvement of type of nasal bleed were having anterior epistaxis i.e. 277 (91.12%) as compared to posterior epistaxis which were only 27 (8.88%) Table 2.

**Table 2:** Distribution of cases acc to side of bleed

Side of Bleed	Gender		Total(%)
	Male	Female	
Right	87	48	135(44.4%)
Left	21	7	28(9.2%)
Bilateral	102	39	141(46.3%)
Total	210	94	304

It was observed that maximum patients were found to have slight bleed and no suctioning required i.e. 145 (47.70%), followed by 138 (45.39%) in whom also slight bleed present with occasional suctioning needed, and least in whom no bleed found i.e.1(0.33%), and severe bleed and constant suctioning required i.e. 1(0.33%).

### Anterior Rhinoscopy

In patients of epistaxis on anterior rhinoscopy, we found deviated nasal septum in highest number of patients i.e. 159 (52.30%) followed by abrasion at little’s area in 110 (36.18%) patients. Deviated nasal septum with spur and nasal discharge seen in 49 (16.12%) patients, foreign body seen in 20 (6.58%), maggots infestation found in 5 (1.65%) and in only 2 (0.66%) patients we found atrophic changes on anterior rhinoscopy as seen in Table 3.

**Table 3:** Anterior rhinoscopy findings

Anterior rhinoscopy findings	Number of patients	Percentage
Abrasion at little’s area	110	36.18%
Nasal discharge	49	16.12%
Nasal mass	4	1.32%
Deviated Nasal Septum	159	52.30%
Inferior turbinate hypertrophy	19	6.25%
DNS with spur	49	16.12%
Atrophic changes	2	0.66%
Maggots	5	1.65%
Foreign body	20	6.58%

### Causes of Epistaxis Observed

According to etiology found in patients of epistaxis, maximum patients were of nose picking i.e. 113 (37.17%) followed by trauma via accident, assault and fall i.e. 77 (25.33%), followed by hypertension i.e. 49 (16.12%) as seen in Table 4. Out of total 304 patients, maximum patients of nose picking were of age group 1–20 years i.e. 84 among 113 patients of nose picking i.e. mainly children while in hypertensive cases, out of total 49 cases, 36 were of age group 41–60 years i.e. mainly adult

**Table 4:** Causes of epistaxis

Etiology	Number of patients	Percentage
LOCAL		
Nose picking	113	37.17%
RTA/assualt/fall	77	25.33%
Foreign body	21	6.91%
Maggots	5	1.64%
DNS with spur	2	0.66%
Infections	12	3.85%
Neoplasms	2	0.66%
GENERAL		
Hypertension	49	16.12%
Infections	6	1.97%
Bleeding disorders	3	0.99%
Drugs	2	0.66%
Chronic alcohol/liver failure	2	0.66%
Vicarious menstruation	1	0.33%
Idiopathic	9	2.96%
Total	304	100.00%

## Discussion

It was found in the study that maximum number of patients with epistaxis were of age group 21–30 years. Epistaxis was found to be more prevalent in the young adults, which is in agreement with Eziyi et al. [7]. But contrary to findings by Pallin et al. [8] who found a bimodal age-related frequency with peaks among those younger than 10 years and aged 70–79 years.

Another author most of their patients to be older than 40 years which correlates with other reports which showed that epistaxis is a geriatric problem [9]. The low age incidence in our study may be attributed to the fact that the majority of our patients had traumatic epistaxis and patients with traumatic epistaxis tended to be younger than those with atraumatic epistaxis.

The epistaxis was found to affect more males than females. The male dominance in this study may be attributed to high incidence of traumatic epistaxis which inclines to affect young males because of their frequent involvement in high risk taking behaviour [9]. 

In our study we found that nose pricking, RTA and hypertension were most prevalent factors causing nasal bleeding. The etiological factors of epistaxis vary from one study to the other but the most prominent three being idiopathic, trauma and hypertension [3, 10]. 

## Conclusion

We conclude that nasal bleeding is most common in younger age group with male predominance .Evaluation of recurrent or severe cases includes a search for underlying causes, such as bleeding disorders and neoplasia. Many techniques, materials, and procedures treat nasal bleeding effectively and sometimes more than one treatment must be used. Otolaryngologists must be prepared to deal with severe or refractory bleeding through the use of medications, packing materials, and radiologic or surgical intervention.
